# Catalytic Enantioselective Aryl Transfer to Aldehydes Using Chiral 2,2’-Bispyrrolidine-Based Salan Ligands

**DOI:** 10.3390/molecules16042971

**Published:** 2011-04-06

**Authors:** Xuefeng Jia, Aijun Lin, Zhijie Mao, Chengjian Zhu, Yixiang Cheng

**Affiliations:** 1 School of Chemistry and Chemical Engineering, State Key Laboratory of Coordination Chemistry, Nanjing University, Nanjing, 210093, China; 2 School of Chemistry and Materials Science, Shanxi Normal University, Linfen, 041004, China; 3 State Key Laboratory of Organometallic Chemistry, Shanghai Institute of Organic Chemistry, Chinese Academy of Sciences, Shanghai, 200032, China; 4 School of Chemistry and Chemical Engineering, Key Laboratory of Mesoscopic Chemistry of MOE, Nanjing University, Nanjing, 210093, China

**Keywords:** 2,2’-bispyrrolidine, salan ligands, aryl transfer, arylboronic acid, enantioselectivity

## Abstract

Chiral *C*_2_-symmetric diamines have emerged as versatile auxiliaries or ligands in numerous asymmetric transformations. Chiral 2,2’-bispyrrolidine-based salan ligands were prepared and applied to the asymmetric aryl transfer to aldehydes with arylboronic acids as the source of transferable aryl groups. The corresponding diarylmethanols were obtained in high yields with moderate to good enantioselectivitives of up to 83% ee.

## 1. Introduction

Chiral diarylmethanols are important intermediates and precursors for the synthesis of pharmacologically and biologically active compounds [1,2,3,4,5,6,7,8]. Therefore, the development of effective catalyst systems for the synthesis of these compounds is of significant importance for organic chemists. The scientifically important protocols for the synthesis of chiral diarylmethanols commonly involve two strategies: (1) the asymmetric reduction of prochiral diaryl ketones [9,10,11,12,13], (2) the enantioselective aryl transfer to aromatic aldehydes [14,15,16]. The reduction method requires an *ortho* substituent on one of the aryls or electronic different aryl groups for optimum results. The second method seems easy to realize chiral induction due to the large steric and electronic differences between an aryl group and a hydrogen atom on the aldehyde substrates with diphenylzinc. As reported previously, many functionalized diarylzincs used as the transferring nucleophiles are unstable and difficult to synthesize, so the method of the aryl transfer to aldehyde is greatly limited. Recently, an elegant method that the arylzinc species prepared *in situ* by transmetalation between organoboron [17,18,19] or organoboronic derivatives [20,21,22,23,24,25,26] and diethylzinc has been proposed as an alternative for the synthesis of salt-free organozinc reagents. We have also successfully developed an efficient and practical method for the synthesis of diarylmethanols by transmetalation using the arylboronic acid in the presence of trimethylgallium [27]. These methods allow the exploitation of a broad range of substituted aryl transfer reagents since numerous arylboronic acids are commercially available, and a lot of excellent ligands were developed and applied to the asymmetric aryl transfer reaction with good results [28,29,30,31,32,33,34,35,36,37,38,39,40,41]. For the future, the introduction of the new, effective and more easily available catalysts is also a field of continuous interest for the catalytic aryl transfer reaction.

Chiral *C*_2_-symmetric diamines have emerged as versatile auxiliaries or ligands in numerous asymmetric transformations [42,43,44]. (*R*,*R*)-2,2’-bispyrrolidine, initially developed by Hirama, was synthesized by various routes [45,46,47,48,49,50], and its derivatives had been successfully employed as chiral ligands or organocatalysts in many asymmetric reactions [51,52,53,54,55,56,57,58,59]. So far, the application of 2,2’-bispyrrolidine-based salan ligands [60,61] in asymmetric catalysis has not been reported. We describe herein our efforts toward the synthesis of optically active diarylmethanols through the asymmetric aryl transfer to aldehydes under the catalysis of (*R*,*R*)-2,2’-bispyrrolidine-based salan ligands.

## 2. Results and Discussion

**Figure 1:**
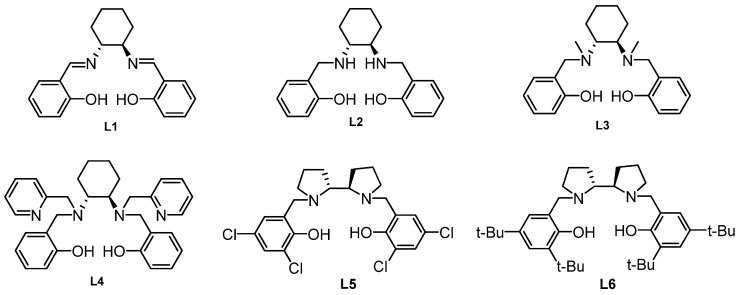
Structures of Ligands L1-L6.

A preliminary study was performed to test the catalytic property of the ligands **L1-L6** (Figure 1) in the asymmetric phenyl transfer reaction to 4-nitrobenzaldehyde at 0 °C. As is evident from Table 1, the resulting products could be obtained in moderate yield, but low enantioselectivity when (1*R*,2*R*)-cyclohexane-1,2-diamine-based ligands **L1-L4** were tested (Table 1, entries 1-4). Gratifyingly, we found that the ligands **L5** and **L6** were more effective in this reaction (Table 1, entries 5-6). The ee value of the product could be increased to 63% when the reaction was carried out at −25 °C (Table 1, entry 7). Increasing catalyst loading had a positive impact on both the yield and enantioselectivity. The best result was obtained in 88% yield with 83% ee while using 20 mol% of **L6** (Table 1, entry 9).

After having established the optimal protocol for the asymmetric phenyl transfer reaction, we further extended the reaction to a series of aldehyde substrates (Table 2). The electronic properties of the aromatic rings of the aldehydes have a significant influence on the enantioselectivity in this reaction. The aldehydes with electron-withdrawing substituents provided better results than those with electron-donating substituents in terms of ee values. 4-Nitrobenzaldehyde gave the corresponding diarylmethanol with 83% ee, but 4-methoxybenzaldehyde only with 11% ee (Table 2, entries 1, 2 and 10). Similar results were obtained when 3-substituted-benzaldehydes (Table 2, entries 3 and 9) or 2-substituted-benzaldehydes (Table 2, entries 5, 6 and 8) were tested. However, an exception was observed for 2-nitrobenzaldehyde (Table 2, entry 4), presumably caused by the chelating effect of the NO_2_ group with the lewis acids [62,63]. The enantioselectivity was also found to be influenced by the steric effect with the same exception of 2-nitrobenzaldehyde. *ortho*-Substituted (-Cl or -Me) benzaldehydes gave higher ee values (Table 2, entries 6 *vs* 2 or 8 *vs* 9). It should be noted that the reaction of 2-naphthaldehyde proceeded well, giving 70% ee and good yield (Table 2, entry 11), and α,β-unsaturated cinnamaldehyde gave the corresponding product with only moderate enantioselectivity (Table 2, entry 12).

We also further investigated the asymmetric aryl transfer to aromatic aldehydes with substituted phenylboronic acids. As shown in Table 3, when 4-chlorophenylboronic acid was chosen as the aryl source and 4-nitrobenzaldehyde as the substrate, 71% ee was obtained (Table 3, entry 1). And 54% ee was obtained when 4-methoxylphenylboronic acid was tested (Table 3, entry 3). 

## 3. Experimental

### 3.1. General

All reactions were carried out under an argon atmosphere using standard Schlenk techniques. Solvents were dried and distilled prior to use according to standard methods. Unless otherwise indicated, all materials were obtained from commercial sources and liquid aldehydes were freshly distilled before use. For thin-layer chromatography (TLC), compounds were visualized by irradiation with UV light on GF 254 silica gel plates. ^1^H-NMR and ^13^C-NMR spectra were recorded in CDCl_3_ on a Bruker ARX-300 spectrometer with chemical shifts being referenced to SiMe_4_ used as internal standard. The coupling constants *J* are given in Hz. HPLC analysis were performed on a chiral column (Daicel Chiralcel OB-H, OD-H or AD-H column) on a Chromatography Interface 600 Series Link instrument and Series 200 pump), with Series 200 UV/VIS detection at 254 nm. The solvent system used has hexane (A)-2-propanol (B) in the indicated proportions. Optical rotations were measured on Rudolph Research Analytical Autopol III Automatic Polarimeter equipped with a 100 mm cell. Mass spectra (EI-MS) were taken using a Shimadzu GCMS-QP2010 mass spectrometer. High Resolution Mass Spectra (HRMS) were taken using a LTQ Orbitrap XL ThermoFisher unit.

### 3.2. Typical Procedure for the Asymmetric Aryl Transfer Reaction

In a 20 mL flame-dried Schlenk reaction tube, diethylzinc (0.9 mmol, 6 equiv, 1.5 M in toluene solution) was added dropwise to a solution of phenylboronic acid (0.3 mmol, 2 equiv) in toluene (3 mL) under an argon atmosphere. After stirring for 12 h at 60 °C, a toluene solution of **L6** (20 mol%) was introduced. The reaction was stirred for an additional 30 minutes and cooled to −25 °C followed by the addition of aldehydes (0.15 mmol). After completion of the reaction (monitored by TLC), the reaction solution was quenched with saturated aqueous NH_4_Cl (3 mL) and further extracted with ethyl acetate (3 × 5 mL). The combined organic layer was dried over Na_2_SO_4_. Evaporation of the solvent gave the crude product, which was further purified by preparative TLC to afford the corresponding chiral diarylmethanols. 

*(S)-4-Nitrophenyl(phenyl)methanol* (**3a**). ^1^H-NMR: δ 8.19 (d, *J =* 7.2 Hz, 2H), 7.58 (d, *J =* 7.2 Hz, 2H), 7.37–7.33 (m, 5H), 5.92 (s, 1H), 2.25 (brs, 1H). 83% ee determined by HPLC with a Chiralcel OB-H column (A/B = 70:30, 0.8 mL/min, uv 230 nm): t_R_ = 21.05 min (minor), t_R_ = 35.74 min (major). [α]_D_^23^ = +31.6 (c = 0.50, EtOH).

*(S)-4-Chlorophenyl(phenyl)methanol* (**3b**). ^1^H-NMR: δ 7.38–7.33 (m, 4H), 7.31–7.27 (m, 5H), 5.80 (s, 1H), 2.20 (brs, 1H). 41% ee determined by HPLC with a Chiralcel OB-H column (A/B = 90:10, 1.0 mL/min, uv 230 nm): t_R_ = 10.21 min (minor), t_R_ = 18.33 min (major). [α]_D_^23^ = +5.9 (*c* = 0.64, EtOH).

*(S)-3-Nitrophenyl(phenyl)methanol* (**3c**). ^1^H-NMR: δ 8.30 (s, 1H), 8.11 (d, *J* = 8.1 Hz, 1H), 7.72 (d, *J* = 7.8 Hz, 1H), 7.50 (t, *J* = 7.8 Hz, 1H), 7.40–7.29 (m, 5H), 5.92 (s, 1H), 2.13 (brs, 1H). 75% ee determined by HPLC with a Chiralcel OB-H column (A/B = 80:20, 0.8 mL/min, uv 230 nm): t_R_ = 34.19 min (minor), t_R_ = 47.40 min (major). [α]_D_^23^ = +42.5 (*c* = 0.40, EtOH).

*(R)-2-Nitrophenyl(phenyl)methanol* (**3d**). ^1^H-NMR: δ 7.94 (dd, *J* = 7.8, 1.5 Hz, 1H), 7.75 (dd, *J* = 7.8, 1.5 Hz, 1H), 7.64 (dt, *J* = 7.5, 1.2 Hz, 1H), 7.46 (t, *J* = 7.8, 1.5 Hz, 1H), 7.36–7.29 (m, 5H), 6.44 (s, 1H), 2.02 (brs, 1H). 41% ee determined by HPLC with a Chiralpark AD-H column (A/B = 90:10, 0.8 mL/min, uv 254 nm): t_R_ = 13.57 min (major), t_R_ = 14.62 min (minor); [α]_D_^23^ = 11.2 (*c* = 0.32, EtOH).

*(R)-2-Trifluoromethylphenyl(phenyl)methanol* (**3e**). ^1^H-NMR: δ 7.66 (t, *J* = 7.8 Hz, 2H), 7.55 (t, *J* = 7.8 Hz, 1H), 7.42–7.32 (m, 5H), 7.30–7.27 (m, 1H), 6.32 (s, 1H), 1.99 (brs, 1H). 80% ee determined by HPLC with a Chiralcel OD-H column (A/B = 90:10, 0.5 mL/min, uv 254 nm): t_R_ = 9.33 min (major), t_R_ = 11.79 min (minor). [α]_D_^23^ = −37.2 (*c* = 0.5, EtOH).

*(S)-2-Chlorophenyl(phenyl)methanol* (**3f**). ^1^H-NMR: δ 7.60 (d, *J* = 7.8 Hz, 1H), 7.42–7.39 (m, 2H), 7.36–7.28 (m, 5H), 7.25–7.22 (m, 1H), 6.24 (s, 1H), 2.05 (brs, 1H). 73% ee determined by HPLC with a Chiralcel OB-H column (A/B = 90:10, 1.0 mL/min, uv 230 nm): t_R_ = 8.97 min (minor), t_R_ = 10.00 min (major). [α]_D_^23^ = −20.6 (*c* = 0.64, EtOH).

*(S)-3-Bromophenyl(phenyl)methanol* (**3g**). ^1^H-NMR: δ 7.57 (s, 1H), 7.42–7.35 (m, 5H), 7.33–7.27 (m, 2H), 7.20 (t, *J* = 7.8 Hz, 1H), 5.78 (s, 1H), 2.33 (brs, 1H). 26% ee determined by HPLC with a Chiralcel OB-H column (A/B = 90:10, 1.0 mL/min, uv 230 nm): t_R_ = 15.17 min (minor), t_R_ = 27.81 min (major). [α]_D_^23^ = +11.4 (*c* = 0.76, EtOH).

*(S)-2-Methylphenyl(phenyl)methanol* (**3h**). ^1^H-NMR: δ 7.53 (d, *J* = 9.0 Hz, 1H), 7.34–7.21(m, 7H), 7.16 (t, *J* = 8.1 Hz, 1H), 6.02 (s, 1H), 2.26 (s, 3H), 1.95 (s, 1H). 60% ee determined by HPLC with a Chiralcel OB-H column (A/B = 90:10, 1.0 mL/min, uv 230 nm): t_R_ = 9.47 min (minor), t_R_ = 10.6 min (major). [α]_D_^23^ = −19.3 (*c* = 0.30, EtOH).

*(S)-3-Methylphenyl(phenyl)methanol* (**3i**). ^1^H-NMR: δ 7.41–7.34 (m, 4H), 7.30–7.27 (m, 2H), 7.24–7.16 (m, 2H), 7.09 (d, *J* = 7.5 Hz, 1H), 5.81 (s, 1H), 2.35 (s, 3H), 2.02 (brs, 1H). 52% ee determined by HPLC with a Chiralcel OB-H column (A/B = 90:10, 1.0 mL/min, uv 230 nm): t_R_ = 12.39 min (minor), t_R_ = 21.34 min (major). [α]_D_^23^ = −15.8 (*c* = 0.34, EtOH).

*(S)-4-Methoxylphenyl(phenyl)methanol* (**3j**). ^1^H-NMR: δ 7.37–7.34 (m, 3H), 7.30–7.26 (m, 4H), 6.88 (d, *J* = 9.0 Hz, 2H), 5.81 (s, 1H), 3.78 (s, 3H), 2.23 (brs, 1H). 11% ee determined by HPLC with a Chiralcel OB-H column (A/B = 90:10, 1.0 mL/min, uv 230 nm): t_R_ = 21.79 min (minor), t_R_ = 24.03 min (major). [α]_D_^23^ = 8.1 (*c* = 0.42, EtOH).

*(S)-2-Naphathyl(phenyl)methanol* (**3k**). ^1^H-NMR: δ 7.90 (s, 1H), 7.86–7.79 (m, 3H), 7.50–7.42 (m, 5H), 7.38–7.28 (m, 3H), 6.01 (s, 1H), 2.06 (brs, 1H). 70% ee determined by HPLC with a Chiralcel OD-H column (A/B = 85:15, 0.8 mL/min, uv 230 nm): t_R_ = 12.71 min (major), t_R_ = 15.08 min (minor). [α]_D_^23^ = −18.4 (c = 0.46, EtOH).

*(R)-1, 3-Diphenylprop-2-en-1-ol* (**3l**). ^1^H-NMR: δ 7.47–7.33 (m, 5H), 7.32–7.27 (m, 3H), 7.29–7.24 (m, 2H), 6.70 (d, *J* = 15.6 Hz, 1H), 6.40 (dd, *J* = 6.3, 15.6 Hz, 1H), 5.40 (d, *J* = 6.6 Hz, 1H), 2.15 (brs, 1H). 47%ee determined by HPLC with a Chiralcel OD-H column (A/B = 80:20, 0.8 mL/min, uv 254 nm): t_R_ = 9.31 min (minor), t_R_ = 11.14 min (major). [α]_D_^20^ = +13.5 (*c* = 0.40, EtOH).

*4-Chlorophenyl(4-nitrophenyl)methanol* (**4a**). ^1^H-NMR: δ 8.20 (d, *J* = 8.7 Hz, 2H), 7.55 (d, *J* = 8.7 Hz, 2H), 7.36–7.27 (m, 4H), 5.90 (s, 1H), 2.04 (brs, H). 71% ee determined by HPLC with a Chiralcel OB-H column (A/B = 80:20, 0.8 mL/min, uv 230 nm): t_R_ = 22.92 min (minor), t_R_ = 25.07 min (major). [α]_D_^23^ = −19.5 (*c* = 0.64, EtOH).

*4-Chlorophenyl(3-bromophenyl)methanol* (**4b**). ^1^H-NMR: δ 7.53 (s, 1H), 7.41 (d, *J* = 7.5 Hz, 1H), 7.39–7.28 (m, 4H), 7.25–7.24 (m, 2H), 7.20 (t, *J* = 7.5 Hz, 1H), 5.77 (s, 1H), 2.04 (brs, 2H). 55% ee determined by HPLC with a Chiralcel OD-H column (A/B = 85:15, 0.8 mL/min, uv 230 nm): t_R_ = 7.80 min (major), t_R_ = 8.58 min (minor). [α]_D_^23^ = +22.8 (*c* = 0.60, EtOH).

*(S)-4-Methoxylphenyl(4-nitrophenyl)methanol* (**4c**). ^1^H-NMR: δ 8.19 (d, *J* = 8.7 Hz, 2H), 7.57 (d, *J* = 8.4 Hz, 2H), 7.25 (d, *J* = 8.4 Hz, 2H), 6.89 (d, *J* = 8.7 Hz, 2H), 5.88 (s, 1H), 3.80 (s, 3H), 2.20 (s, 1H). 54%ee determined by HPLC with a Chiralpark AD-H column (A/B = 85:15, 0.8 mL/min, uv 254 nm): t_R_ = 15.60 min (minor), t_R_ = 19.25 min (major). [α]_D_^23^ = +27.9 (*c* = 0.44, EtOH).

*4-Methoxylphenyl(3-nitrophenyl)methanol* (**4d**). ^1^H-NMR: δ 8.28 (s, 1H), 8.10 (d, *J* = 8.1 Hz, 1H), 7.71 (d, *J* = 8.1 Hz, 1H), 7.49 (t, *J* = 8.1 Hz, 1H), 7.27 (d, *J* = 6.6 Hz, 2H), 6.89 (d, *J* = 6.9 Hz, 2H), 5.88 (s, 1H), 3.80 (s, 3H), 2.28 (brs, 1H). 24% ee determined by HPLC with a Chiralcel OD-H column (A/B = 85:15, 0.8 mL/min, uv 230 nm): t_R_ =15.08 min (major), t_R_ =16.23 min (minor). [α]_D_^23^ = +23.8 (*c* = 0.50, EtOH).

*3, 5-Dimethylphenyl(phenyl)methanol* (**4e**). ^1^H-NMR: δ 7.42–7.28 (m, 5H), 7.01 (s, 2H), 6.93 (s, 1H), 5.77 (s, 1H), 2.31 (s, 6H), 2.18 (brs, 1H). 48% ee determined by HPLC with a Chiralcel OD-H column (A/B = 90:10, 0.8 mL/min, uv 254 nm): t_R_ = 8.67 min (minor), t_R_ = 9.58 min (major). [α]_D_^23^ = +20.4 (*c* = 0.65, EtOH).

## 4. Conclusions

In summary, we haved reported the asymmetric aryl transfer to aldehydes with arylboronic acids as aryl sources in the presence of the chiral 2,2’-bispyrrolidine-based ligand **L6**. The corresponding diarylmethanols could be obtained in high yields with moderate to good enantioselectivities. Further work on the asymmetric addition mechanism and the broad application of chiral 2,2’-bispyrrolidine-based ligands in other asymmetric catalytic reactions are now in progress in our laboratory.

## Figures and Tables

**Table 1:**
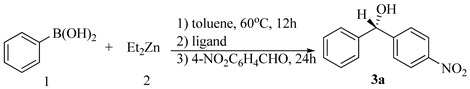
Asymmetric Phenyl Transfer to 4-nitrobenzaldehyde. *^a^*

Entry	Ligand	Mol%	T(°C)	Yield(%)^b^	Ee(%)*^c^*
1	L1	10	0	66	6
2	L2	10	0	73	11
3	L3	10	0	69	16
4	L4	10	0	80	3
5	L5	10	0	74	31
6	L6	10	0	84	43
7	L6	10	-25	70	63
8	L6	15	-25	80	71
9	L6	20	-25	88	83(*S*) *^d^*

*^a^* All the reactions were carried out on 0.2 mmol scale of substrates with 2 equiv of arylboronic acid and 6 equiv of Et_2_Zn in toluene for 24 h. *^b^* Isolated yields. *^c^* Determined by HPLC with a Chiralcel OB-H column. *^d^* The absolute configuration of the products were determined by comparison with literature values.

**Table 2:**
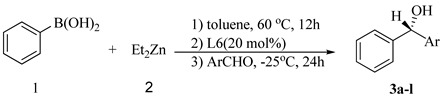
Asymmetric Phenyl Transfer to Aromatic Aldehydes. *^a^*

Entry	Ar	Product	Yield (%)*^b^*	Ee (%)*^c,d^*
1	4-NO_2_C_6_H_4_	**3a**	88	83(*S*)
2	4-ClC_6_H_4_	**3b**	80	41(*S*)
3	3-NO_2_C_6_H_4_	**3c**	91	75(*S*)
4	2-NO_2_C_6_H_4_	**3d**	85	41(*R*)
5	2-CF_3_C_6_H_4_	**3e**	72	80(*R*)
6	2-ClC_6_H_4_	**3f**	84	73(*S*)
7	3-BrC_6_H_4_	**3g**	85	26(*S*)
8	2-MeC_6_H_4_	**3h**	76	60(*S*)
9	3-MeC_6_H_4_	**3i**	78	52(*S*)
10	4-MeOC_6_H_4_	**3j**	80	11(*S*)
11	2-C_10_H_7_	**3k**	80	70(*S*)
12	PhCH=CH	**3l**	76	47(*R*)

**Table 3:**
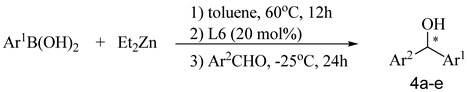
Asymmetric Aryl Transfer to Aldehydes. *^a^*

Entry	Ar^1^	Ar^2^	Product	Yield (%)*^b^*	Ee (%)*^c^*
1	4-Cl C_6_H_4_	4-NO_2_C_6_H_4_	4a	75	71
2	4-Cl C_6_H_4_	3-BrC_6_H_4_	4b	82	55
3	4-MeOC_6_H_4_	4-NO_2_C_6_H_4_	4c	78	54(*S*)*^d^*
4	4-MeOC_6_H_4_	3-NO_2_C_6_H_4_	4d	70	24
5	3,5-diMeC_6_H_3_	C_6_H_5_	4e	70	48

*^a^* All reactions were carried out on 0.15 mmol scale of substrates with 2 equiv of arylboronic acid and 6 equiv of Et_2_Zn in toluene at −25 °C for 24 h in the presence of 20 mol% ligand; *^b^* Isolated yields; *^c^* Enantiomeric excess was determined by HPLC with a Chiralcel OB-H, OD-H or AD-H column; *^d^* The absolute configuration of the products were determined by comparison with literature values.
